# Toxicity and Immunogenicity of Enterotoxigenic *Escherichia coli* Heat-Labile and Heat-Stable Toxoid Fusion 3xSTa_A14Q_-LT_S63K/R192G/L211A_ in a Murine Model

**DOI:** 10.1371/journal.pone.0077386

**Published:** 2013-10-11

**Authors:** Chengxian Zhang, David E. Knudsen, Mei Liu, Donald C. Robertson, Weiping Zhang, the STa Toxoid Vaccine Consortium Group

**Affiliations:** 1 Veterinary & Biomedical Sciences Department /The Center for Infectious Disease Research & Vaccinology, South Dakota State University, Brookings, South Dakota, United States of America; 2 Department of Diagnostic Medicine/Pathobiology, Kansas State University, Manhattan, Kansas, United States of America; KIIT University, India

## Abstract

Diarrhea is the second leading cause of death to young children. Enterotoxigenic Escherichia coli (ETEC) are the most common bacteria causing diarrhea. Adhesins and enterotoxins are the virulence determinants in ETEC diarrhea. Adhesins mediate bacterial attachment and colonization, and enterotoxins including heat-labile (LT) and heat-stable type Ib toxin (STa) disrupt fluid homeostasis in host cells that leads to fluid hyper-secretion and diarrhea. Thus, adhesins and enterotoxins have been primarily targeted in ETEC vaccine development. A recent study reported toxoid fusions with STa toxoid (STa_P13F_) fused at the N- or C-terminus, or inside the A subunit of LT_R192G_ elicited neutralizing antitoxin antibodies, and suggested application of toxoid fusions in ETEC vaccine development (Liu et al., Infect. Immun. 79:4002-4009, 2011). In this study, we generated a different STa toxoid (STa_A14Q_) and a triple-mutant LT toxoid (LT_S63K/R192G/L211A_, tmLT), constructed a toxoid fusion (3xSTa_A14Q_-tmLT) that carried 3 copies of STa_A14Q_ for further facilitation of anti-STa immunogenicity, and assessed antigen safety and immunogenicity in a murine model to explore its potential for ETEC vaccine development. Mice immunized with this fusion antigen showed no adverse effects, and developed antitoxin antibodies particularly through the IP route. Anti-LT antibodies were detected and were shown neutralizing against CT *in vitro*. Anti-STa antibodies were also detected in the immunized mice, and serum from the IP immunized mice neutralized STa toxin *in vitro*. Data from this study indicated that toxoid fusion 3xSTa_A14Q_-tmLT is safe and can induce neutralizing antitoxin antibodies, and provided helpful information for vaccine development against ETEC diarrhea.

## Introduction

 Diarrhea is the second leading cause of death to young children who live in developing countries [1], and continues to be a major threat to global health [2]. Enterotoxigenic Escherichia coli (ETEC), *E. coli* strains producing enterotoxins, are the most common bacteria that cause diarrhea, and are responsible for 300,000 - 500,000 deaths of young children annually [2,3]. In addition, ETEC strains are the most common cause of diarrhea to children and adults travelling to ETEC endemic countries or regions, military personnel deployed at these areas, and immunocompromised patients [2,4-6]. These ETEC strains produce various bacterial adhesins and one or more enterotoxins. Bacterial adhesins mediate ETEC initial attachment to host epithelial cells and subsequent colonization at host small intestines, and 23 different adhesins including colonization factor antigens (CFAs) and coli surface antigens (CSs) were characterized among ETEC strains [7]. Enterotoxins including heat-labile toxin (LT) and heat-stable toxin type Ib (STa) disrupt fluid homeostasis in host small intestinal epithelial cells to cause hyper-secretion of electrolyte-rich fluid through activation of intracellular adenylate cyclase (by LT) or guanylate cyclase (by STa), that leads to diarrhea [8]. Since being identified as virulence determinants in ETEC-associated diarrhea, adhesins and toxins have been primarily targeted in anti-adhesin and antitoxin vaccine development. It is believed that anti-adhesin vaccines inducing immunity to block attachment and colonization of ETEC at host small intestines, and antitoxin vaccines inducing antitoxin immunity to neutralize LT and STa enterotoxicity should provide effective protection against ETEC diarrhea [9,10]. Unfortunately, there are no effective vaccines currently available to protect against ETEC diarrhea [10], despite the facts that the association between *E. coli* and children diarrhea was discovered over 100 years ago [11], that the disease mechanism of ETEC-associated diarrhea has been well studied [8,10], and that ETEC strains have been identified the leading bacteria that cause diarrhea [2]. 

 Developing broadly effective ETEC vaccines is hampered by challenges including heterogeneity of ETEC adhesins and potent toxicity of enterotoxins. As different ETEC strains produce immunologically heterogeneous adhesins, experimental vaccines targeting on one adhesin provide protection against only ETEC expressing the same or homologous adhesin, but not strains expressing heterogeneous adhesins. The potent enterotoxicity of LT and STa pre-excludes both toxins from being considered as antigens in developing safe vaccines. Moreover, STa, a 19-amino-acid peptide, is poorly immunogenic, thus itself cannot be used as a vaccine component [10,12,13]. In addition, STa shares no genetic or antigenic homology with LT; therefore, anti-LT immunity is not cross protective against STa toxin. Indeed, early experimental vaccines using LT antigens (the nontoxic LT_B_ subunit) were found protective against only ETEC strains expressing LT toxin but not against strains expressing STa toxin [14,15]. Now it becomes acknowledged that an effective antitoxin vaccine should include both LT and STa antigens to induce anti-LT and anti-STa immunity. To be included as safe vaccine components, however, LT and STa would first have their toxicity eliminated or reduced, and only STa and LT derivatives with toxicity reduced or eliminated can be considered safe antigens; second, STa must also have its immunogenicity facilitated to stimulate anti-STa immune responses [16,17]. STa peptides were found to induce anti-STa antibodies when genetically fused or chemically conjugated to strongly immunogenic carrier proteins, such as BSA or detoxified LT peptides [15,17-21]. Recently, studies demonstrated that some full-length non-toxic STa molecules can be genetically fused to a detoxified LT toxoid (LT_R192G_) and resultant LT-STa toxoid fusions were found safe and elicited neutralizing antibodies against both toxins, and suggested that LT-STa toxoid fusions can be potentially used for developing antitoxin vaccines against ETEC diarrhea [17,22,23]. 

 In this study, we generated a different STa molecule, STa_A14Q_, and a less toxic triple-mutant LT, LT_S63K/R192G/L211A_ (tmLT), to construct a different toxoid fusion antigen. STa_A14Q_ was selected because its analogue, porcine-type pSTa_A13Q_ not only has toxicity more reduced but also maintains an antigenic topology more similar to native STa toxin compared to toxoids pSTa_N11K_ (an analogue of STa_N12K_) and pSTa_P12F_ (an analogue of STa_P13F_) [17]. Therefore, this STa_A14Q_ toxoid, after being genetically fused to an LT toxoid, is expected to elicit stronger neutralizing antibodies against STa toxin. Attempting to further facilitate anti-STa immunogenicity, we genetically fused three copies of STa_A14Q_ at the N-terminus, the C-terminus, and inside the LT_A_ subunit of tmLT. This constructed toxoid fusion was evaluated in a murine model for safety and immunogenicity, and potential application in ETEC vaccine development.

## Materials and Methods

### Bacterial strains and plasmids

**Figure 1:**
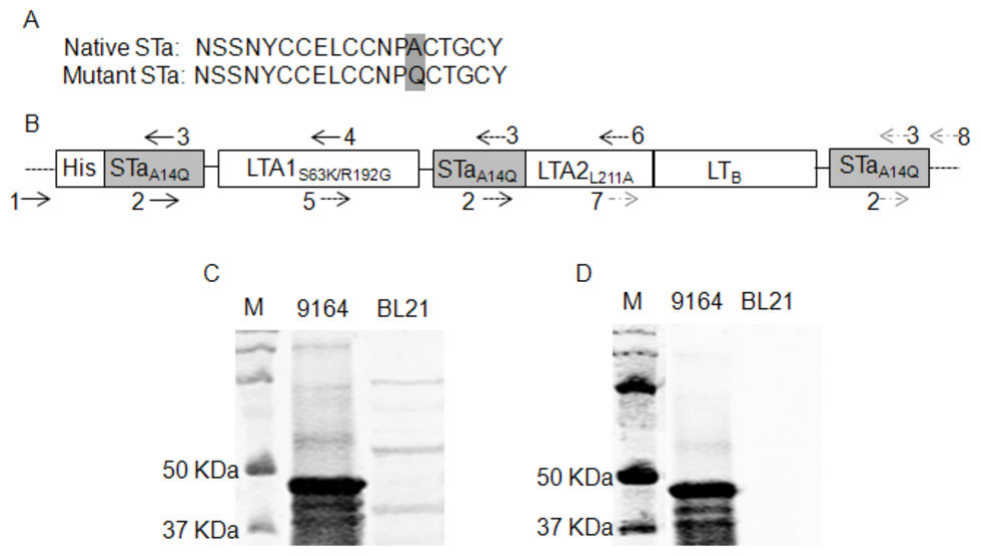
Construction and detection of the 3xSTa_A14Q_-tmLT toxoid fusion. Panel A: Amino acid sequences of the native STa toxin and the STa_A14Q_ toxoid. Panel B: Construction of the 3xSTa_A14Q_-tmLT toxoid fusion gene with 3 copies of the STa_A14Q_ toxoid gene genetically fused at the 5’ end, within LT_A_, and the 3’ end of the tmLT toxoid gene (LT_S63K/R192G/L211A_). The numbers and arrows indicated primers used in PCRs to mutate the genes and to amplify fragments to be overlapped for a single open reading frame encoding the 3xSTa_A14Q_-tmLT toxoid fusion. This chimeric toxoid fusion gene was cloned in vector pET28α and expressed in *E. coli* BL21 as a 6xHis-tagged fusion protein. The drawing scale is not in proportion to nucleotide fragment sizes. Panel C: Detection of 6xHis-tagged 3xSTa_A14Q_-tmLT toxoid fusion protein in Western blot using 12% PAGE gel, with rabbit anti-CT antiserum (1:3300; Sigma) and IRDye-labeled goat anti-rabbit IgG (1:5000; LI-COR, Lincoln, NE). Panel D: Detection of the toxoid fusion protein with purified rabbit anti-STa antiserum (1:5000) and IRDye-labeled goat anti-rabbit IgG (1:5000; LI-COR, Lincoln, NE). Extracted 6xHis-tagged protein form fusion strain 9164 and total protein extracts from negative control strain 8955 were examined in the SDS-PAGE. Lane M is the protein marker (Precision Plus Protein Pre-stained standards, Bio-Rad, Hercules, CA).

*E. coli* strains and plasmids used in this study are listed in Table 1. Three previously constructed LT_R192G_-STa_P13F_ toxoid fusion recombinant strains: 8751, 8752 and 8753 [22], were used as templates to construct the new fusion strain. Since the *eltAB* genes (coding LT_AB_ toxin) and the *estA* gene (coding heat-stable toxin 1b; Figure 1A) were of human ETEC prototype strain H10407, the *eltAB*, LT, *estA*, and STa in this study are of the human-type. *E. coli* TOP10 (Invitrogen, Carlsbad, CA) and BL21 (GE Healthcare, Piscataway, NJ) were used as host strains. Vector pBR322 (Promega, Madison, WI) was used to clone the mutated STa and LT genes, and pET28α (Novagen, Madison, WI) was used to clone and express the toxoid fusion gene. Strain 8955, a BL21 strain with vector pET28α [23], was used as the negative control. Recombinant *E. coli* strains were cultured in Luria Broth (LB) supplemented with ampicillin (100 µg/ml) or kanamycin (30 µg/ml).

**Table 1:** *Escherichia coli* strains and plasmids used in the study.

Strains	Relevant properties	Plasmid	Reference
BL21	*B F* ^*-*^ *, omp*T, *hsd*S (r_B_^-^, m_B_^-^), *gal*, *dcm.*		GE Healthcare
8751	LT_R192G_-STa_P13F_, BL21/pfusion-2b	LT_192_-L-STa_13_/pET28α	[22]
8752	STa_P13F_-LT_R192G_, BL21/pfusion-3b	STa_13_-gly-pro-LT_192_/pET28α	[22]
8753	LT_R192GA1_-STa_P13F_-LT_A2-B_, BL21/pfusion-4b	LT_192A1_-gly-pro-STa_13_-LT_A2-B_/pET28α	[22]
TOP10	F^-^ *mcr*AΔ(*mrr*-*hsd*RMS-*mcr*BC) Φ80*lac*ZΔM15 Δ*lac*X74 recA1 *deo*R *ara*D139Δ(*ara*-*leu*)7697 *gal*U *gal*K *rps*L (Str^R^)endA1 *nup*G		Invitrogen
8325	STa recombinant strain, TOP10	STa in pBR322	[23]
8407	pSTa_A14Q_ mutant strain, TOP10	STa_A14Q_ in pBR322	this study
8460	LT recombinant strain, TOP10	LT in pBR322	[22]
8543	LT_R192G_ mutant strain, TOP10	LT_R192G_ in pBR322	[22]
9123	LT_S63K_ mutant strain, TOP10	LT_S63K_ in pBR322	this study
9125	LT_S63K/R192G_ mutant strain, TOP10	LT_S63K/R192G_ in pBR322	this study
9078	LT_R192G/L211A_ mutant strain, TOP10	LT_R192G/L211A_ in pBR322	this study
9127	LT_S62K/R192G/L211A_ mutant strain, TOP10	LT_S62K/R192G/L211A_ in pBR322	this study
9157	p3xSTa_A14Q_-tmLT plasmid^*^, TOP10	3xSTa_A14Q_-tmLT in pET28α	this study
9164	p9157, BL21	3xSTa_A14Q_-tmLT in pET28α	this study
8955	Negative control, BL21/pET28α	pET28α	[23]

*: Nucleotides coding the transmembrane signal peptides of the mutated *eltA*, *eltB* and *estA* genes, and the stop codons of the *eltA, eltB*, & the firs 2 copies of *estA* were removed to construct the fusion as a single open reading frame.

### Cloning and mutation of the LT and STa genes, and construction of the toxoid fusion gene

The STa gene (*estA*), that was isolated from *E. coli* H10407 and cloned into pBR322 [22,23], was mutated at nucleotides coding the 14^th^ amino acid (GCT to CAG) for mutant STa_A14Q_ (Figure 1A). Two PCR amplified products: one using primers pBRNheI-F [22] and hSTa14Q-R (5’-cccggtacactgaggattacaaca’-3), the other with primers hSTa14Q-F (5’tgttgtaatcctcagtgtaccggg-‘3) and pBREagI-R [22], were overlapped in a splicing overlap extension (SOE) PCR. The overlapped fragment was digested with NheI and EagI and cloned into vector pBR322 for STa mutant STa_A14Q_. Similar to the PCR method previously used to mutate the 192^th^ amino acid (AGA to GGA) for toxoid LT_R192G_ [17,22], native *eltAB* genes isolated from *E. coli* H10407 were mutated at the 211^th^ amino acid (CTC to GCC) using primers LT211-F (5-‘cagaatctgagcacaatatatgccag-‘3; nucleotides underlined were the target mutation) and LT211-R (5’-tgattgatatttcctggcatatattgt-‘3) for mutant LT_L211A_, at the 63^th^ (TCT to AAA) with primers LT63-F (5’-gtttccactaaacttagtttgagaagt -‘3) and LT63-R (5’-caaactaagtttagtggaaacatatc-‘3) for mutant LT_S63K_, at both the 192^th^ and the 211^th^ for double-mutant LT_R192G/A211L_ (dmLT), and at the 63th, 192^th^ and the 211^th^ for triple-mutant LT_S63K/R192G/L211A_ (tmLT). All mutated *eltAB* genes were cloned into pBR322 vector and expressed in *E. coli* TOP 10 cells. Expression and secretion of the mutated STa and LT were examined in STa competitive ELISA and GM1 ELISA as described previously [17].

 To construct the toxoid fusion gene carrying 3 copies of the STa_A14Q_ gene and 1 copy of the tmLT gene, we genetically fused together 3 pieces of DNA fragments, which were resulted from 3 SOEs, to form a single open reading frame (Figure 1B). Briefly, the first piece of fragment consisted of nucleotides coding the first copy of STa_A14Q_ and the first 63 amino acids of the LT_A1_ peptide. That came from an overlap of two PCR products: one by primer 1 (T7-F, 5’-taatacgactcactataggg-‘3) paired with primer 3 (hSTa14Q-R), and the other by primer 2 (STa14Q-F) paired with primer 4 (LT63-R), with plasmid of 8752 (STa_13_-gly-pro-LT_192_/pET28α) as the template. The second piece of fragment contained nucleotides coding the second copy of STa_A14Q_ and the LT_A_ peptide coding the 64 - 211 amino acids. This fragment was generated by the overlap of another two PCR products: one by primer 5 (LT63-F) with primer 3 (hSTa14Q-R) and the other by primer 2 (hSTa14Q-F) with primer 6 (LT211-R), with plasmid 8753 (LT_192A1_-gly-pro-STa_13_-LT_A2-B_/pET28α) as the DNA template. The third fragment included the LT_A_ peptide coding the 212-240 amino acids, the LT_B_ peptide, and a third copy of STa_A14Q_ (with a stop codon). The third fragment was resulted from the overlap of PCR products using primer 7 (LT211-F) and primer 3 (hSTa14Q-R), and primer 2 (hSTa14Q-F) paired with primer 8 (T7-R, 5’-tgctagttattggtcaggggt-‘3), with plasmid 8751 (LT_192_-L-STa_13_/pET28α) as the template. The second and third fragments were connected first in another SOE PCR, and the resultant fragment was further overlapped to the first fragment in a final SOE PCR to generate the single-open-reading-frame toxoid fusion gene. Since the DNA templates (p8751,8752,p8753) used in PCRs derived from strain 8543 that had nucleotides coding the LT_192_ residue mutated, and PCR primers carried mutated nucleotides coding the LT_63_ and LT_211_ residues, overlapping these 3 pieces of fragments resulted in an entire chimeric toxoid fusion gene designated as 3xSTa_A14Q_-LT_S63K/R192GL211A_ (Figure 1B). All PCRs and SOEs were carried out with *pfu* DNA polymerase (Strategene/Agilent Technologies, Santa Clara, CA) as described previously [17,22]. The final overlapped fragment was further amplified with PCR primers T7-F and T7-R, digested with NheI and EagI restriction enzymes, cloned into pET28α vector, and expressed as a 6xHis-tagged protein in *E. coli* TOP10 or *E. coli* BL21, by following standard protocols [17,22,24].

### Expression and detection of the 3xSTa_A14Q_-tmLT toxoid fusion protein

Expression of the 6xHis-tagged fusion protein by *E. coli* TOP10 and BL21 recombinant strains was examined in a standard sodium dodecyl sulfate-polyacrylamide gel electrophoresis (SDS-PAGE). The toxoid fusion recombinant strain was grown at 37 °C in 500 ml LB medium supplemented with kanamycin (30 µg/ml), and was induced with isopropyl-1-thio-β-D-galactoside (IPTG; 100 µM) for 4 h after culture optical density (OD) reached 0.5. Bacteria were pelleted with centrifugation and were resuspended with 5 ml bacterial protein extraction reagent (B-PER, in phosphate buffer; Pierce, Rockford, IL) for total protein extraction (largely from inclusion body, in denatured buffer), followed by extraction of 6xHis-tagged proteins to a purity of greater than 90% using nickel affinity chromatography with Ni-nitrilotriacetic acid (Ni-NTA) agarose by following the manufacturer’s protocol (QIAGen, Valencia, CA). Extracted 6xHis-tagged proteins were refolded using a Pierce^®^ Protein Refolding kit with #5 and #9 buffers (Thermo Scientific, Rochester, NY). Refolded proteins were dialyzed 24 h at 4 °C in a series of guanidine-HCl (Sigma, St. Louis, MO) -PBS buffers with guanidine concentrations gradually reduced from 6M to 1M by following manufacture’s recommendation (Thermo Scientific), then concentrated using Spectra/Por^®^ molecularporous membrane tubing (Spectrum Laboratories Inc., Rancho Dominquez, CA) and polyethylene glycol compound (PEG; Sigma) at 4 °C overnight. Ten microliter 6xHis-tagged protein extracts were analyzed in a 12% SDS-PAGE gel and immuno-blot assay. Rabbit anti-CT (1:3300; Sigma) and anti-STa antisera (1:5000; Robertson laboratory) were used to detect the fusion protein. IRDye-labeled goat anti-rabbit IgG (1:5000; LI-COR, Lincoln, NE) were used as the secondary antibody. Bound proteins were detected using a LI-COR Odyssey premium infrared gel imaging system (LI-COR). In addition, ELISAs using fusion proteins as the coating antigen and anti-CT and anti-STa antiserum as antibodies were carried out to assess the toxoid fusion for LT and STa antigenicity, and GM_1_ ELISA to examine its binding activity to GM_1_ receptors.

### Toxicity detection of the STa toxoid, LT toxoids, and the 3xSTa_A14Q_-tmLT toxoid fusion protein

Toxicity of the expressed STa_A14Q_, LT_S63K_, LT_S63K/R192G_, LT_R192G/L211A_ and LT_S63K/R192G/L211A_ proteins (in pBR322 vector and *E. coli* TOP10 cells), with prototype H10407 and STa recombinant strain 8325 as positives, as well as the 3xSTa_A14Q_-tmLT fusion protein (200 ng or 2 µg) with purified STa and CT toxins as references, was assessed in T84 cells using EIA cGMP and cAMP kits (Assay Designs, Ann Arbor, MI). As described previously [17,22], 150 µl overnight-grown culture supernatants (from an equal amount of cells based on culture optical density values) of each toxoid mutant strain and the positive control strain, the extracted refolded fusion protein (200 ng or 2 µg in 150 µl PBS), 10 ng cholera toxin (CT, Sigma; in 150 µl PBS) which is highly homologous to LT structurally and functionally, or 2 ng purified STa toxin (from Robertson laboratory; in 150 µl PBS), were added to each microplate well (in duplicate) that had 1x10^5^ T-84 cells seeded. After 2 h incubation in a CO_2_ incubator, wells were washed and T-84 cells were lysed. The lyses were collected and used in ELISAs to measure intracellular cAMP or cGMP levels (pmol/ml) by following the manufacturer’s protocol.

### Mouse immunization with toxoid fusion protein

A group of 8 to 10 female adult BALB/c mice (Charles River Laboratories International, Inc., Wilmington, MI) were immunized intraperitoneally (IP) or intranasally (IN) with the refolded 3xSTa_A14Q_-tmLT toxoid fusion protein. For IP immunization, each of the 10 mice in the immunization group was IP injected with 100 µl of the refolded fusion protein (1.3 mg/ml) and 100 µl Freund’s complete adjuvant (Sigma), and each of the 10 mice in the control group was injected with 100 µl Freund’s complete adjuvant and 100 µl 0.02 M Tris-HCl buffer. Two booster injections at the same dose but with Freund’s incomplete adjuvant were followed biweekly. Mice for IN immunization were divided into 4 groups: the first group of 10 mice was each administrated with 30 µl of the refolded fusion protein (1.3 mg/ml) mixed with 2 µg CT (as adjuvant), the second group of 6 mice was given the adjuvant alone (2 µg CT in 30 µl 0.02 M Tris-HCl buffer), the third group of 8 mice was given 30 µl of the refolded fusion protein (1.3 mg/ml) without adjuvant, and the fourth group of 8 mice received the 0.02 M Tris-HCl buffer only. All samples were administrated drop by drop (4-5 µl) alternatively to each mouse nostril cavity with a p-100 pipettor and a flat flexible gel loading pipettor tip. Mice were held at a horizontal position for 1 min after each drop so that antigen could have a prolong stay at nostril area. The same dose was given at each booster on day 14 and day 28. Mice were anaesthetized with CO_2_ and exsanguinated at day 37. Serum and fecal samples (1 g feces resuspended in 5 ml fecal reconstitution buffer; 1:6 dilution) [22,25] collected before and 7 days after each immunization were stored at -80 °C until use. After exsanguination mice were necropsied, and intestines were collected and minced with a pair of surgical scissors in PBS (1 g in 2.5 ml; 1:3.5 dilution) supplemented with protease inhibitor phenylmethylsulfonyl fluoride (0.2 mg/ml). Minced products were vortexed for 1 min and centrifuged 3 min at 13,000 rpm to collect supernatants. Collected supernatants were referred as ‘intestinal wash samples’ in this study. Animal studies were in compliance with the Animal Welfare Act by following The 1996 National Research Council guidelines [26], and were approved and supervised by a state veterinarian and the South Dakota State University’s Institutional Animal Care and Use Committee.

### Antitoxin antibody titration

Anti-LT and anti-STa IgG and IgA antibodies in serum, fecal suspension and intestine wash samples of each mouse were examined in ELISAs. ELISAs were conducted similarly as described previously [17,22,23], but with modification. In ELISA to titrate anti-LT antibodies, 25 ng CT (an LT homologue which has been commonly used as coating antigen to titrate anti-LT antibodies) in 100 µl antigen coating buffer (0.015 M Na_2_CO_3_, 0.035 M NaHCO_3_, pH9.6) were coated to each well of an Immulon 2HB plate (Thermo Scientific, Rochester, NY) for 1 h at 37 °C and followed by overnight at 4 °C. Coated wells were washed with PBST (0.05% Tween-20), and uncoated sites were blocked with 10% non-fat milk-PBST (150 µl per well) for 1 h at 37 °C. After washing (5x with PBST), each well was incubated with serum (initially diluted 1:200 in 5% milk-PBST), fecal suspension (1:20 dilution in 5% milk-PBST), or intestine wash sample (1:25 dilution in 5% milk-PBST), in a binary dilution, for 1 h at 37 °C. Wells were washed (5x with PBST) and incubated with HRP-conjugated goat anti-mouse IgG (1:5000 dilution; Sigma) or IgA (1:1000 dilution; Sigma), 100 µl per well, for 1 h at 37 °C; then washed again (3x with PBST and 2x with PBS), and incubated with TMB Microwell Peroxidase Substrate System (2-C) (KPL, Gaithersburg, MD), 100 µl per well, for 20 min at room temperature. Optical density (OD) was measured with a plate reader at 405 nm wavelength. OD readings, greater than 0.4 after subtraction of background readings, were calculated for antibody titers at a scale of log_10_, as described previously [17,22].

 In ELISA to titrate anti-STa IgG and IgA antibodies, 1.3 - 2 ng STa-ovalbumin conjugates (from D. C. Robertson Laboratory), in 100 µl STa ELISA buffer [27], were added to each well of a Costar plate (Corning Inc., Corning, NY) and incubated for 1 h at 37 °C and followed by overnight at 4 °C. After washing twice with PBST, each well was blocked with 150 µl 5% non-fat milk (in PBST) at 37 °C for 1 h. After three washes, 100 µl serum (1:25 dilution in 1% milk-PBST), fecal suspension 1:15-20 diluted (in 1% milk-PBST) or without diluting, or intestine wash samples (1:10-15 dilution in 1% milk-PBST) was added to each well, binarily diluted, and incubated 1 h at 37 °C. After washing (4x), each well had 100 µl HRP-conjugated goat-anti-mouse IgG (1:3000 dilution) or IgA (1:1000 dilution) antibodies added and incubated for 1 h at 37 °C; followed by washes (3x with PBST and 2x with PBS) and incubation with 100 µl TMB peroxidase substrate (KPL) for 20 min. OD measurement and antibody titration were conducted the same as described above.

 Samples in anti-LT antibody titration ELISAs were examined in triplicate, and in anti-STa antibody titration ELISAs were in duplicate due to limited supply of the STa-ovalbumin conjugates. Each antibody titration ELISA was repeated at least once. Weekly collected serum and fecal samples, of individual mouse or pooled from the group, were also used to measure anti-LT and anti-STa antibody titers.

### Antitoxin antibody neutralization assays

Mouse serum, fecal suspension and intestine wash samples were examined for antibody neutralization activities against CT and STa toxins using T-84 cells and EIA cAMP and cGMP kits (Assay Design). Since neutralizing antitoxin antibodies prevent CT or STa from stimulating intracellular cyclic GMP or AMP levels in T-84 cells, antibody neutralization activities against CT (or LT) and STa toxins can be assessed with cyclic GMP and cAMP ELISA kits. As described previously [17], serum (30 µl serum in 120 µl DMEM/F12 medium; 1:33.3 dilution in a final volume of 1 ml), fecal (30 µl 1:6 diluted fecal suspension in 120 µl DMEM/F12 medium, a 1:200 final dilution; or 150 µl 1:6 diluted fecal suspension, a 1:40 dilution) and intestine washes (30 µl 1:2.5 diluted washes in 120 µl DMEM/F12 medium; 1:83.3 final dilution), pooled from each group or from individual mouse, were incubated with 2 ng STa toxin or 10 ng CT (diluted in 150-µl DMEM/F12 medium). After 1 h at room temperature, the mixture (300 µl in total) was added to T-84 cells (with 700 µl culture medium), and incubated 2 h at 37 °C in a CO_2_ incubator. Cells were washed and lysed, and cell lysates were collected and measured for intracellular cAMP or cGMP levels (pmol/ml) using cAMP and cGMP ELISA kits, respectively.

### Statistical analysis

Data were analyzed by using the SAS for windows version 8 (SAS Institute, Cary, N.C.), adjusting for multiple comparison by Bonferroni. Results were expressed as means ± standard errors. Student’s *t*-test was used to compare the different treatment groups. Calculated p values of < 0.05 were regarded as significant when treatments were compared at two-tailed distribution and two-sample equal or unequal variance. 

## Results

### STa_A14Q_ and tmLT (LT_S63K/R192G/L211A_) showed significant reduction in toxicity

Cloning of the mutated STa or LT genes in each mutant strain was verified from DNA sequencing, and expression and secretion of the STa and LT proteins were confirmed from STa competitive ELISA and GM1 ELISA, respectively. *In vitro* toxicity assays indicated that the mutated STa and LT had toxicity eliminated or reduced. Cyclic GMP ELISA showed the cGMP level (to measure STa toxicity) in T-84 cells incubated with culture supernatant of STa_A14Q_ mutant strain 8407 (Table 1) was 0.093 pmole/ml, a level significantly lower (p=0.007, p<0.001) compared to the cGMP levels in the T-84 cells incubated with the culture supernatant of STa recombinant strain 8325 (1.42 pmol/ml) or wildtype strain H10407 (2.79 pmol/ml). Similar to STa toxoids, modified LT also showed toxicity reduction. The cAMP levels (to measure LT toxicity) in the T-84 cells incubated with culture supernatant of 9123 (LT_S63K_), 9125 (LT_S63K/R192G_), 9078 (LT_R192G/L211A_) and 9127 (LT_S63K/R192GL211A_) (Table 1) were 3.55, 3.35, 6.45 and 2.5 pmole/ml, respectively. These levels were significantly lower compared to the cAMP level in the T-84 cells incubated with the culture supernatant of LT recombinant strain 8460 (16.3 pmole/ml; p=0.008, 0.002, 0.034, 0.013).

### The 3xSTa_A14Q_-tmLT toxoid fusion protein was expressed

DNA sequencing verified that the toxoid gene consisted of 3 copies of the mutated STa gene (*estA*) which carried no precursor sequences and only the mutated STa at the 3’end of the fusion gene carried nucleotides coding the stop codon, and one copy of a triple mutated LT_S63K/R192G/L211A_ (Figure 1B). It was also revealed that the entire toxoid fusion was a single open reading frame. Expression of this 3xSTa_A14Q_-tmLT fusion protein in strain 9164 was confirmed in Western blot. A protein at the size close to 50 KDa, equivalent to the expected size of the monomeric 3xSTa_A14Q_-tmLT fusion, was detected with rabbit anti-CT (Figure 1C) and anti-STa antiserum (Figure 1D). No proteins of 50 KDa from the *E. coli* BL21 host strain 8955 were detected. Additionally, ELISAs indicated that this fusion protein reacted to anti-CT antiserum, with an average OD value of 0.58 (1.69 in wells coated with CT; 0.079 in negative control wells). The GM_1_ ELISA showed this fusion protein had significant reduction in binding to GM_1_ receptor, compared to CT (OD values 0.19 vs. 1.33, p < 0.001).

### The 3xSTa_A14Q_-tmLT toxoid fusion protein was shown to be safe *in vitro*

**Figure 2:**
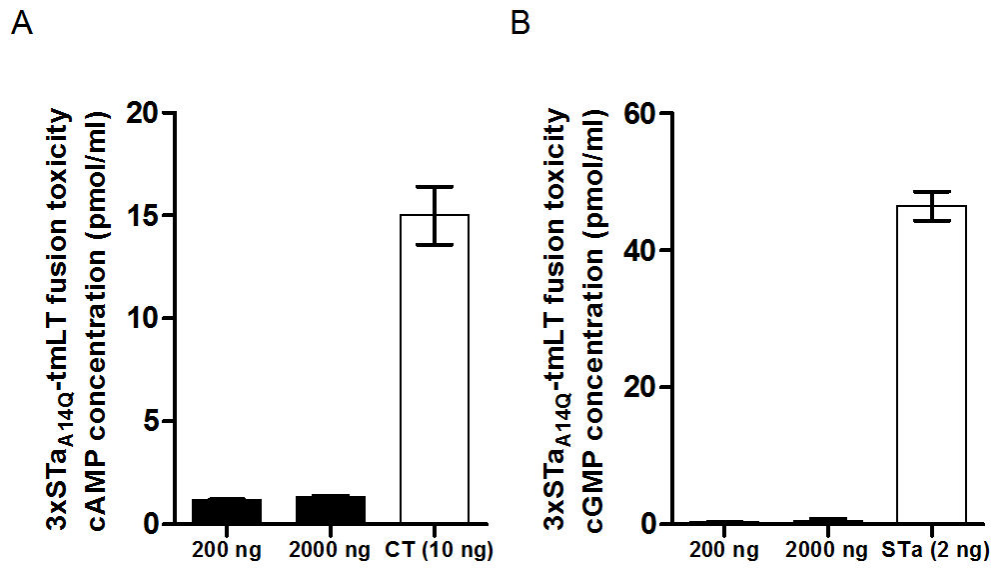
Fusion protein toxicity assays in T-84 cells using cAMP and cGMP EIA ELISA kits. 200 ng or 2 µg of the refolded toxoid fusion protein 3xSTa_A14Q_-tmLT were incubated with T-84 cells, and intracellular cAMP (panel A) and cGMP (panel B) levels (pmol/ml) were measured. T-84 cells incubated with the toxoid fusion stimulated significant less cAMP (p<0.01) or cGMP (p<0.01) compared to those incubated with 2 ng STa or 10 ng CT.

This 3xSTa_A14Q_-tmLT toxoid fusion protein did not stimulate an increase of cAMP or cGMP in T-84 cells (Figure 2). The cAMP levels in T-84 cells incubated with 200 ng and 2 µg toxoid fusion protein were 1.17 and 1.35 pmole/ml, respectively. These levels were similar to the cAMP level in T-84 cells incubated with cell culture medium alone, but significantly lower compared to the cAMP in the T-84 cells incubated with 10 ng CT (15 pmole/ml; p=0.003, 0.003) (Figure 2A). The cGMP levels in T-84 cells incubated with 200 ng and 2 µg purified toxoid fusion protein were 0.365 and 0.585 pmole/ml, respectively. These levels were not different from the cGMP in T-84 cells treated with cell culture medium (0.38 pmol/ml), but differed significantly from the cGMP in T-84 cells treated with 2 ng STa toxin (46.5 pmole/ml; p<0.001, <0.001) (Figure 2B). Moreover, mice tolerated this fusion protein well, as the immunized mice exhibited no apparent adverse effects.

### Toxoid fusion protein 3xSTa_A14Q_-tmLT was immunogenic

**Figure 3:**
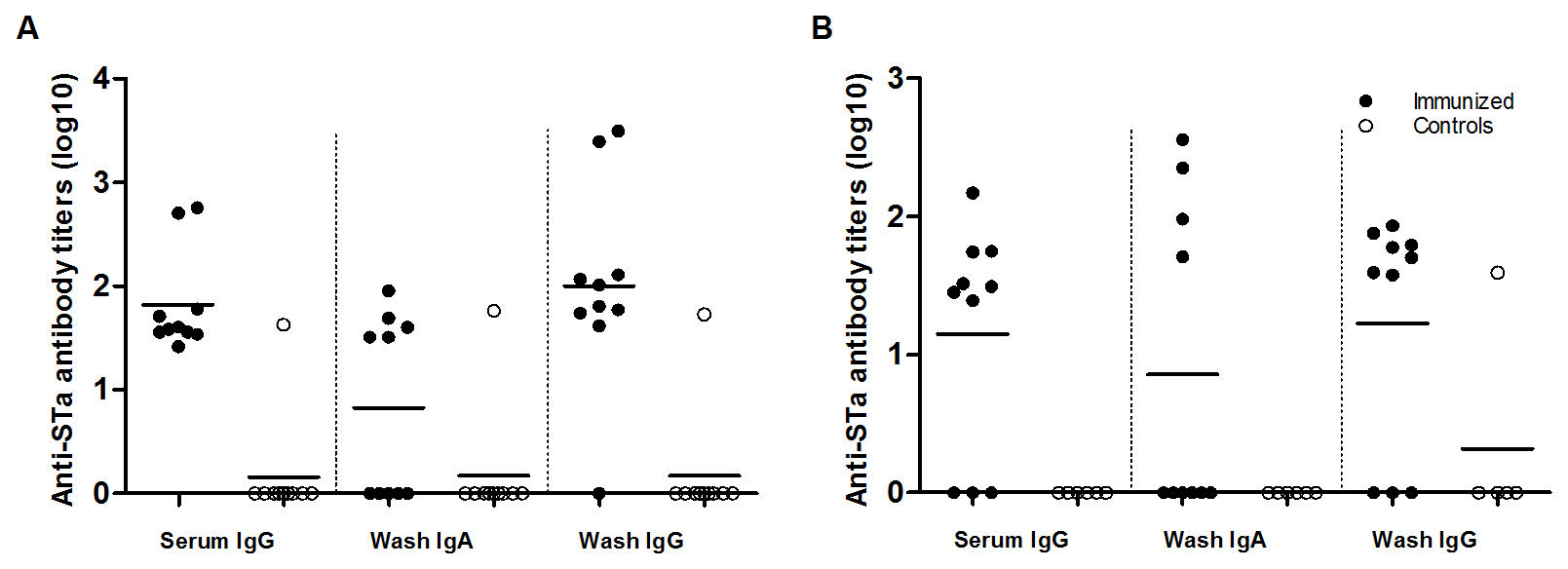
Anti-STa IgG and IgA antibody titration of serum and intestinal wash samples of IP or IN immunized mice (solid dots) and the control mice (circles). Panel A: anti-STa IgG and IgA antibodies titers in the serum and intestine wash samples of the IP immunized mice, but not in the control mice (p<0.01). Panel B: anti-STa IgG and IgA antibodies titers from serum and intestine wash samples of the IN immunized mice, and no anti-STa IgG or IgA antibodies detected in the control mice (p<0.01). For STa antibody titration ELISAs, 1.3 - 2 ng STa-ovalbumin conjugates were used to coat each well of a Costar plate (Nunc); HRP-conjugated goat-anti-mouse IgG (1:3000) or IgA (1:1000) as the secondary antibodies. Optical densities of greater than 0.4 (after subtracting the background reading) were used to calculate anti-STa antibody titers (in log_10_).

Mice IP or IN immunized with the toxoid fusion protein (with adjuvants) had anti-STa antibodies detected. The IP immunized mice had anti-STa IgG antibody detected in the serum samples of all 10 immunized mice and in intestine wash samples of 9 (out 10) immunized mice, and anti-STa IgA antibodies in the intestine wash samples of 5 immunized mice (Figure 3A). Anti-STa IgA antibodies were also examined from the fecal suspension samples, but the ODs were lower than 0.4, the cutoff point; therefore, ELISA data were not included in final analyses. In the IN groups, anti-STa IgG antibodies were detected in the serum (7 out 10 immunized mice) and washes (7 out 10), and anti-STa IgA antibodies only in the intestinal washes (4 out 10) of the mice immunized with the toxoid fusion antigen with CT adjuvant (Figure 3B). When weekly-collected serum samples were examined, anti-STa IgG antibodies were detected after the IP primary immunization and after the first IN booster immunization.

**Figure 4:**
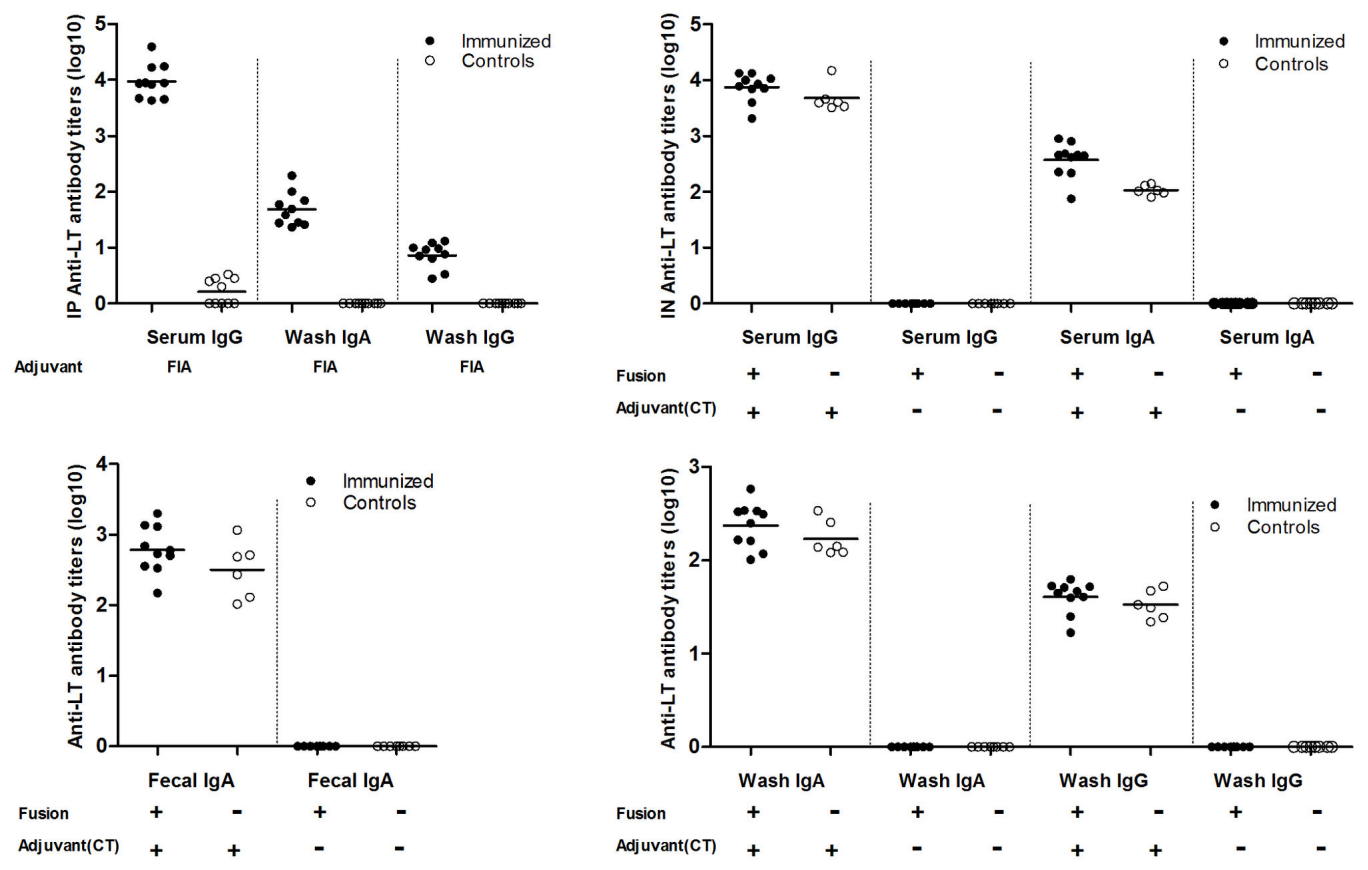
Anti-LT antibody titration from serum, fecal suspension, and intestine wash samples of the IP and IN immunized mice. Panel A: anti-LT IgG and IgA antibody titers from serum and intestine wash samples of the IP immunized (solid dots) and control mice (circles) (p<0.01). Panel B: anti-LT IgG and IgA antibody titers from serum samples of the mice IN immunized with 130 µg fusion antigen with 2 µg CT adjuvant or 2 µg CT adjuvant alone, but not of mice immunized with 130 µg fusion antigen without CT or Tris-HCl buffer only. Panel C: anti-LT IgA antibody titers detected from fecal suspension samples of mice IN immunized with the fusion antigen with 2 µg CT adjuvant or CT adjuvant alone, but not of mice immunized with fusion antigen without CT or Tris-HCl buffer. Panel D: anti-LT IgG and IgA antibody titers detected in intestinal wash samples of the mice IN immunized with the fusion and CT or CT, but not with the fusion alone or Tri-HCl buffer. Anti-LT antibody titration ELISAs used 25 ng CT (Sigma) to coat each well of an Immulon 2HB plate (Thermo Scientific); HRP-conjugated goat-anti-mouse IgG (1:5000) or IgA (1:1000) as the secondary antibodies. Optical densities of greater than 0.4 (after subtracting the background reading) were used to calculate anti-LT antibody titers (in log_10_).

 Anti-LT antibodies were detected in the immunized (with adjuvants) mice (Figure 4). In the IP immunization groups, anti-LT IgG antibodies in the serum and intestinal wash samples, and anti-LT IgA antibodies in the intestinal wash samples were detected from the immunized mice, but not the control mice (Figure 4A). In the IN immunized mice, anti-LT antibodies were detected in mice immunized with either the toxoid fusion antigen (with CT adjuvant) or CT adjuvant alone, but not among mice immunized with the toxoid fusion protein alone or the buffer. Mice IN immunized with the toxoid fusion with CT adjuvant or CT adjuvant alone had anti-LT IgG and IgA antibodies detected in the serum samples (Figure 4B), anti-LT IgA antibodies in the fecal suspension sample (Figure 4C), and anti-LT IgG and IgA in the intestinal wash samples (Figure 4D). Analyses of the weekly collected samples (pooled) showed that IP immunized mice has anti-LT IgG antibodies detected in serum samples after the IP primary immunization and that antibody induction was boosted after each booster immunization. Mice IN immunized with the toxoid fusion (with CT adjuvant) or CT adjuvant had anti-LT antibodies detected in serum samples after the primary injection, and anti-LT IgA detected in the serum samples and fecal suspension after the 1^st^ booster injection. 

### Elicited antibodies neutralized CT and STa toxins *in vitro*

**Figure 5:**
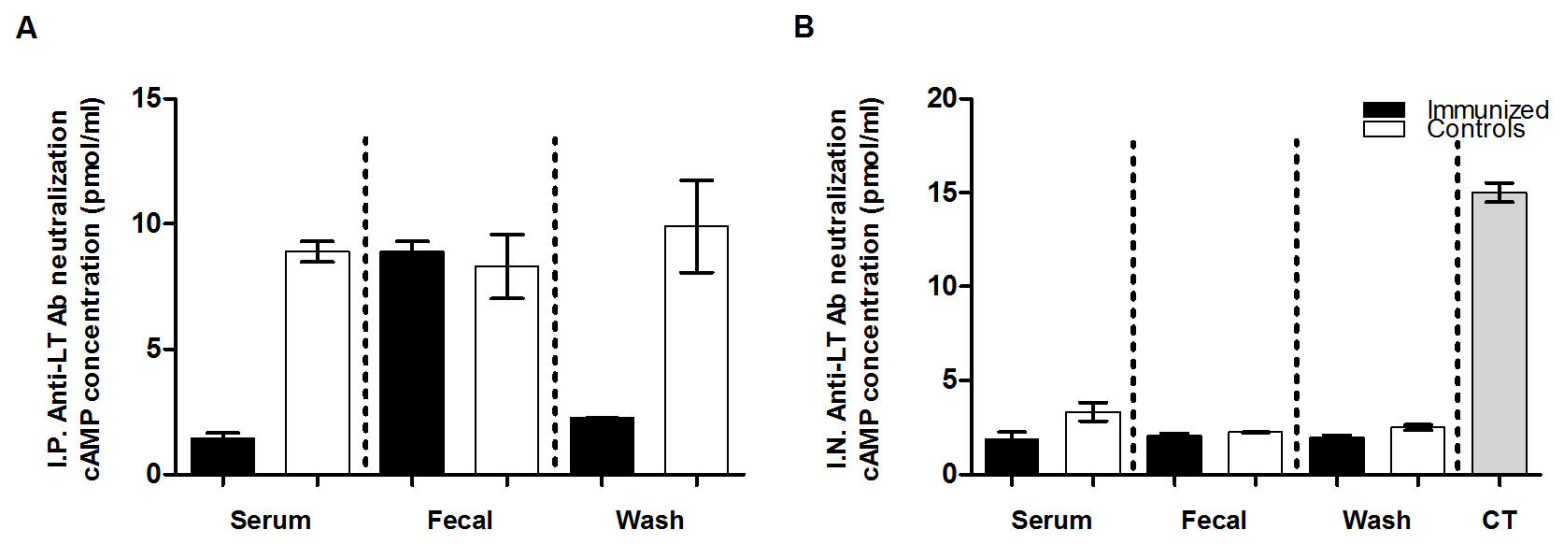
Neutralization against CT from antibodies in pooled serum (1:33.3 dilution), fecal (1:200 dilution) and intestine washes (1:83.3 dilution) of the IP and IN immunized mice. Panel A: serum, fecal suspension and intestine wash samples of the IP immunized mice (solid boxes) and the control mice (open boxes) were examined for neutralizing against 10 ng CT in T-84 cells using EIA cyclic AMP ELISA kit (cAMP EIA, Assay Design). Panel B: serum, fecal suspension and intestine wash samples of mice IN immunized with the 3xSTa_A14Q_-tmLT toxoid fusion with CT (solid boxes) or CT adjuvant (open boxes) were examined for neutralization against 10 ng CT. Intracellular cAMP concentrations (pmol/ml) were measured by following the manufacture’s protocol. Boxes and error bars indicate means and standard deviations.

**Figure 6:**
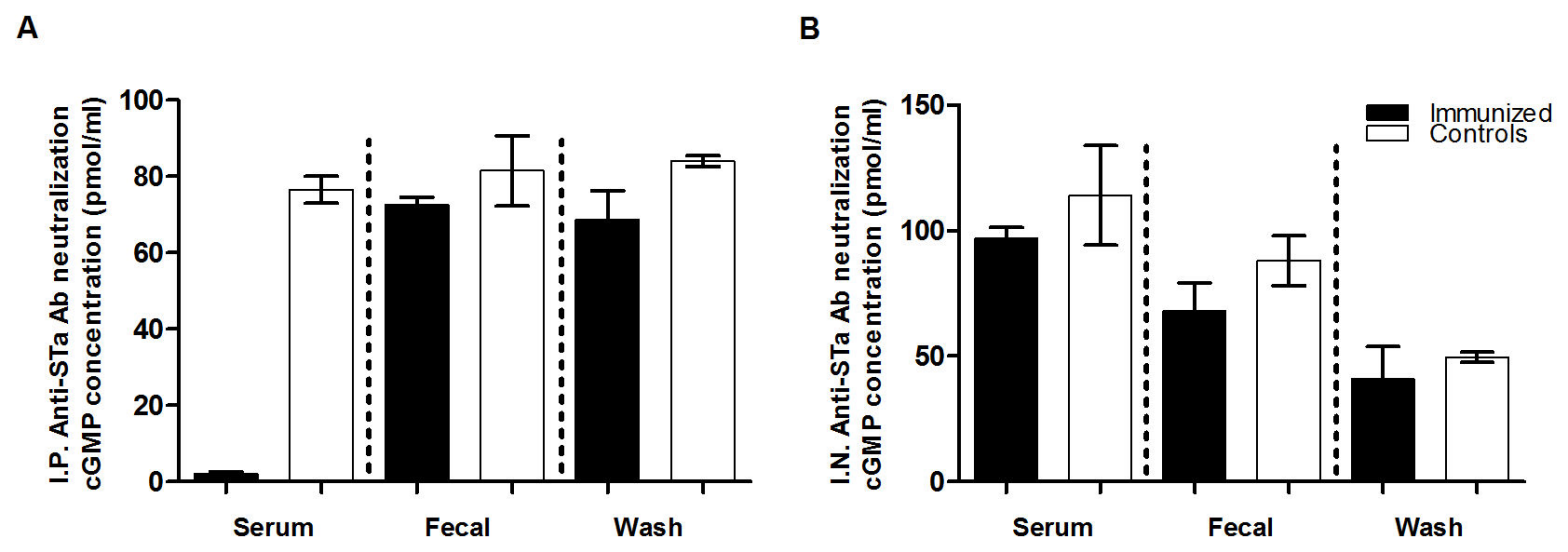
Neutralization against STa toxin from antibodies in pooled serum (1:33.3), fecal suspension (1:200) and intestine washes (1:83.3) of the IP and IN immunized mice. Panel A: serum, fecal suspension and intestine wash samples of the IP immunized mice (solid boxes) and control mice (open boxes) were examined for neutralization against 2 ng STa in T-84 cells using EIA cyclic GMP ELISA kit (cGMP EIA, Assay Design). Panel B: serum, fecal suspension and intestine wash samples of mice IN immunized with the 3xSTa_A14Q_-tmLT toxoid fusion antigen with CT (solid boxes) or CT adjuvant (open boxes) were examined for neutralization against 2 ng STa. Boxes and error bars indicate means and standard deviations.

*In vitro* antibody neutralization assays indicated that elicited antibodies exhibited neutralization activities against CT and STa toxins (Figures 5&6). Cyclic AMP ELISA showed that cAMP levels in the T-84 cells incubated with 10 ng CT and the serum or washes of the IP immunized mice were 1.45 and 2.26 pmole/ml (Figure 5A). These levels were significantly lower than the cAMP in T-84 cells incubated with the same amount of CT but serum (8.9 pmole/ml, p=0.002) or washes (9.9 pmole/ml, p=0.027) from the IP control mice. When incubated with the fecal samples of the IP immunized and control mice, the T-84 cells had the cAMP detected at levels of 8.9 and 8.3 pmole/ml, with no statistically significant differences (p=0.59). But when undiluted fecal suspension samples were used, T-84 cells incubated with fecal suspension of the IP immunized mice had significant lower cAMP level compared to the cells incubated with fecal samples of the control mice ( p=0.02). Incubated with CT (10 ng) and the serum, fecal suspension, or intestinal wash samples from the mice IN immunized with the toxoid fusion (with CT adjuvant), T-84 cells had cAMP levels of 1.88, 2.1, and 1.95 pmole/ml, respectively (Figure 5B). These levels were lower than that in cells treated with the samples from mice immunized with the CT adjuvant alone, as the cAMP levels in T-84 cells incubated with the serum, fecal and washes of the mice IN immunized with CT alone were 3.35, 2.26 and 2.5 pmole/ml. Differences at inhibiting CT in stimulating cAMP in T-84 cells by the serum (p=0.08), fecal (p=0.18), or washes (p=0.06) samples between these two groups (IN with fusion and CT adjuvant vs. IN with CT alone) were not statistically significant. These levels, however, were significantly lower compared to those in cells treated with 10 ng CT alone (15 pmole/ml; p<0.01), or the cAMP levels in cells incubated with CT and serum (p<0.01), fecal suspension (p<0.01) or intestinal wash samples (p<0.01) of the mice IN immunized with either the toxoid fusion without CT adjuvant or the Tris-HCl buffer.

 Elicited antibodies in the serum of the IP immunized mice exhibited neutralizing activities against STa toxin (Figure 6). The cGMP in the T-84 cells incubated with STa toxin and the serum of the IP mice was 2.15 pmole/ml, that was significantly different from the cGMP in cells incubated with STa toxin and the serum sample from the IP control mice (76.5 pmole/ml; p<0.001) (Figure 6A). The cGMP in T-84 cells incubated with fecal suspension or intestinal wash samples of the IP immunized mice were lower than those in cells incubated with fecal and washes of the IP control mice, but were not significantly different (p=0.31, 0.11). When the intestinal wash sample pooled from the 5 IP immunized mice that had anti-STa IgA detected was used, the cGMP in T-84 cells was significantly lower than that in the cells incubated with the intestinal washes of the IP control mice (p=0.04). T-84 cells incubated with STa toxin and the serum, fecal suspension and intestinal washes of the mice IN immunized with the toxoid fusion antigen (with CT adjuvant) had lower cGMP levels compared to cells incubated with the toxin and the serum, fecal suspension and intestinal wash samples from the mice IN immunized with CT alone (Figure 6B), but differences were not statistically significant.

 Serum samples from individual IP immunized mice were examined in neutralization assays against STa toxin. The serum of mouse 7E, among those had greater anti-STa IgG antibody titers detected, showed strong neutralizing activity against STa toxin. It was found that even when 5 µl (versus 30 µl used in the pooled samples) serum was used, STa toxin showed significant reduction in stimulation of intracellular cGMP in T-84 cells (p=0.03). When 10 µl serum (1:100 in final dilution) was used, it completely prevented STa toxin from stimulating any increase of intracellular cGMP in T-84 cells (1.79 pmol/ml, a level detected in T-84 cells incubated with cell culture medium alone).

## Discussion

 As *E. coli* strains producing LT, STa (heat-stable toxin I), or both toxins are the most common bacteria causing diarrhea, effective prevention against ETEC is urgently needed. Vaccines that induce host anti-adhesin immunity to inhibit ETEC attachment and colonization and antitoxin immunity to neutralize enterotoxicity are expected to provide effective protection against ETEC diarrhea. Although significant progress has been made in developing vaccines against colonization from the most prevalent CFA adhesins and against LT enterotoxicity [9,10,12,13], ETEC vaccine candidates currently under development including the promising ones do not carry STa antigens to induce immunity protecting against STa toxin [10]. As ETEC strains expressing STa toxin, alone or together with LT, are associated with two thirds of ETEC-associated diarrhea cases [28,29] and the severe cases [30], only vaccines that also induce anti-STa immunity can provide effective protection against ETEC diarrhea. But the potent toxicity and poor immunogenicity of STa have brought major challenges. It was suggested that STa needs to have its toxicity eliminated or reduced and also anti-STa immunogenicity enhanced in order to be used as an ETEC vaccine antigen [16,17]. Recent studies reported that STa toxoids, such as STa_P13F_, STa_E8A_, STa_T16Q_, STa_G17S_, and porcine-type pSTa_N11K_ and pSTa_P12F_ which are homologues to human-type STa_N12K_ and STa_P13F_, when genetically fused to a full-length LT toxoid (LT_R192G_), induced anti-STa antibodies in mice, rabbits or pigs [17,22,23]. Moreover, elicited antibodies demonstrated neutralizing activity against STa toxin (and CT as well) *in vitro* [17,22], and protected suckling piglets against infection from a STa-producing ETEC strain [17]. The current study demonstrated that the STa_A14Q_ had toxicity eliminated and induced anti-STa antibodies after being genetically fused to an LT antigen, and suggested potential application of toxoid fusion antigens in ETEC vaccine development.

 In this study, we selected STa_A14Q_ to be fused to the LT toxoid and to explore this fusion for eliciting neutralizing anti-STa antibodies. This STa_A14Q_ likely maintains the native STa antigenic epitope topology, indicated by results from a previous study showing its analogue pSTa_A13Q_ (porcine-type STa toxoid) was less toxic but maintained an antigenic structure more similar to native pSTa than toxoid pSTa_N11K_ or pST_P12F_ did [17]. That suggests STa_A14Q_ could be a better candidate for constructing a toxoid fusion antigen to elicit antibodies exhibiting greater neutralizing activity against STa toxin, although its STa antigenic topology could potentially alter after being fused to an LT toxoid. Results from this study showed that antibodies in serum sample of the IP immunized mice neutralized STa toxin, demonstrated by complete prevention of STa toxin in stimulation of any increase of cGMP in T-84 cells (Figure 6A). Future antibody titration using a serial of diluted samples will determine the anti-STa antibody neutralization titer. Assuming that an antigen dose effect would occur, we included 3 copies of STa_A14Q_ in the fusion in order to further facilitate anti-STa immunogenicity in this study. Results from a comparative study, by re-analyzing serum sample from the previous study with the anti-STa antibody titration method used in this study, indicated that mice IP immunized with 3xSTa_A14Q_-tmLT developed anti-STa IgG (serum) titers more than twice greater than the mice previously IP immunized with the LT_R192G_-STa_P13F_ fusion, although a lineal dosage association was not observed (data not shown). That indicated that additional copies of STa toxoid enhanced a toxoid fusion in stimulation of anti-STa immunity and provided helpful information in future ETEC toxoid vaccine development.

 It was noted that antibodies induced by the current 3xSTa_A14Q_-tmLT and the previous LT_R192G_-STa_P13F_ toxoid fusions showed variation regarding neutralizing activities. Strong anti-STa neutralization activity was observed in serum, but poor in fecal or intestinal wash samples, of the mice IP immunized with the current toxoid fusion 3xSTa_A14Q_-tmLT. Results from the previous study suggested that antibodies in fecal samples of mice immunized with fusion LT_192_-STa_P13F_ showed fair to moderate neutralization activity against STa [22]. We also observed that individual mice responded variously to the toxoid fusion antigen. Some IP immunized mice had anti-STa antibody titers detected twice greater than others, and serum sample of these mice, even at a dilution of 1:100 or 1:200, strongly neutralized STa toxin as they prevented STa from stimulation of cGMP in T-84 cells. The structure, or more importantly the antigenic epitope topology, of the STa toxoid presented by toxoid fusions would affect induction of neutralizing anti-STa antibodies. But the cause of variation in immune responses among mice immunized with the same antigen is unclear momently. Immunogenicity of STa toxoids, even enhanced after fused to a strongly immunogenic carrier protein, could be still relatively moderate. That could attribute to inconsistence towards interactions between the STa antigenic domain and host cells, and likely induction of host immune responses in individual mice. Future studies to comparatively examine toxoid fusions, instead of STa toxoids (as STa toxoid topology could be altered after being fused to an LT toxoid), for affinity (or reactivity) to antibodies against native STa can help us to identify fusion antigens with maximum anti-STa immunogenicity to induce antibodies strongly neutralizing against native STa. It should be noted that neutralizing activities against STa (and CT) from antibodies elicited by this toxoid fusion was only assessed *in vitro*, future *in vivo* studies including infant suckling mouse assays and piglet challenge studies will be needed to better evaluate elicited anti-STa antibodies.

 Data from this study indicated that STa_A14Q_ is a good candidate for LT-STa toxoid fusion antigen to elicit neutralizing anti-STa antibodies. But they may also suggest that other modified STa molecules, such as those having mutation at amino acids other than the 8^th^, 12^th^, 13^th^, 14^th^, 16^th^ and 17^th^, or mutations at the same position but with alternative amino acid replacements, may also become STa toxoids and elicit neutralizing anti-STa antibodies when fused to an LT toxoid or other protein carriers. Constructing a STa toxoid or STa-LT toxoid fusion library and thoroughly screening these candidates for not only toxicity reduction but also retention of native STa antigenic structure or topology, a project currently carried out by the STa Toxoid Vaccine Consortium group, may help us to identify optimal STa toxoids for genetic fusions or chemical conjugations to elicit strongly protective anti-STa antibodies.

 The tmLT was used to construct the toxoid fusion antigen in this study because it was less toxic based on the results from intracellular cAMP ELISA assays in T-84 cells, although the cAMP level in T-84 cells incubated with tmLT was not significantly different than those incubated with LT_S63K_ (p=0.24) or LT_S63K/R192G_ (p=0.21), but significantly different compared to dmLT (p=0.046). No studies were carried out to directly compare toxicity of 3xSTa_A14Q_-tmLT with 3xSTa_A14Q_-dmLT or 3xSTa_A14Q_-LT_R192G_ in this study. It is likely that none of these fusions retain LT toxicity, especially considering that only the monomeric LT molecule was presented in these fusions. As toxoid fusion 3xSTa_A14Q_-tmLT did not stimulate an increase of either cAMP or cGMP level in T-84 cells, and mice showed no adverse effects after immunization, this fusion can be considered a safe antigen. Results from IP immunization studies indicated this tmLT maintained LT immunogenicity as neutralizing anti-LT antibodies were developed in mice immunized with the toxoid fusion (with Freund’s adjuvant), thus tmLT is suitable to construct genetic fusions or chemical conjugates for inducing anti-LT immunity and enhancing anti-STa immunity. IN immunization studies, however, showed that mice immunized with this toxoid fusion antigen alone did not stimulate detectable immune responses to LT or STa, unless CT adjuvant was included. That could be caused by the fact that this monomeric toxoid fusion antigen no longer formed a pentamer LT_B_ structure and did not bind effectively to host ganglioside (GM) receptors as LT_B_ pentamer or LT holotoxin did. Binding to GM receptors at host epithelial cells is believed to enhance antigens uptake and stimulation of host mucosal immune responses. On the other hand, binding to gangliosides and then retrograde neuronal transportation of IN administrated LT toxoid LT_S63K_ were believed to cause the undesirable Bell’s palsy to human volunteers [31]. Since it does not effectively bind to ganglioside GM_1_, this toxoid fusion antigen may not cause Bell’s palsy if taken intranasally by human volunteers, but would induce anti-LT and anti-STa immunity at the presence of a proper adjuvant. That may provide helpful information to solve the dilemma in developing IN immunized ETEC vaccines.

 CT (2µg) was used as adjuvant for IN immunization in this study. CT was commonly used as an adjuvant in oral, intragastric and epicutaneous immunization [32-36], as well as in IN immunization [37-39]. Data from this study suggested that CT is a very effective adjuvant for IN immunization, as mice IN immunized with the toxoid fusion with CT developed strong immune responses to LT and STa toxins, whereas those immunized with the toxoid fusion alone did not. Unfortunately, due to the high homology between CT and LT, we were unable to differentiate immune responses induced by the LT of the fusion toxoid or the CT adjuvant. Future studies using alternative adjuvants, like monophosphoryl lipid A (MPL), flagellin or others, instead of CT, may help us to better assess induction of anti-LT immunity by this toxoid fusion antigen through the IN route. On the other hand, because of their homology, anti-CT or anti-LT immunity is cross protective; therefore, immunity, whether induced by the toxoid fusion or CT adjuvant, can protect against ETEC LT toxin. CT was also used as the coating antigen to titrate anti-LT antibodies or the toxin to examine anti-LT antibody neutralization activity in this study. The structure homology between CT and LT made the commercially available CT commonly used for anti-LT antibody titration. Although LT is recently also commercially available, CT is still preferred for *in vitro* anti-LT antibody neutralization assays as CT is more potent in stimulating cAMP levels in T-84 cells.

 This toxoid fusion antigen was also sublingually (SL) administrated to a group of 10 mice. However, in contrast to early studies that showed mice developed strong systemic and mucosal immunity after being SL immunized with LT or CT antigens [40-44], mice SL immunized with toxoid fusion 3xSTa_A14Q_-tmLT (with CT adjuvant) had only anti-STa and anti-LT IgA in the intestine washes, and anti-LT IgG in serum detected. In addition, only antibodies in the serum samples were found to neutralize CT toxin (data not shown). However, in this study, SL administration was carried out without anesthesia, which likely resulted in that the given fusion antigen (40 µg) being swallowed by the mice and thus not effectively delivered to sublingual glands. Under anesthesia, mice would have had antigens effectively delivered and had the given antigens a prolonged stay at sublingual areas to induce robust immune responses. Future studies to SL immunize mice under anesthesia will better assess immunogenicity of this toxoid fusion antigen through the SL route.

 We modified antibody titration ELISA assays in this study, with more stringent blocking, additional washes and use of different microtiter plates (for anti-LT), to eliminate background readings. In the previous study [22], antibody titration data from intestinal wash samples had to be excluded due to background readings. Stringent blocking and additional wash steps clearly reduced ELISA background readings. But on the other hand, the stringent blocking and washing procedures likely resulted in low or no detection of anti-STa and anti-LT antibodies from the fecal suspension samples, and also the lack of detection of anti-STa antibodies in some immunized mice. The nature of low antibodies presented in fecal samples may directly attributed to a low detection of anti-STa antibodies in the fecal samples of the immunized mice. Future studies performing kinetic ELISA instead of endpoint measurement may help to detect immune responses from the fecal suspension samples. Unexpectedly, anti-STa antibodies were detected from one mouse in the IP control group (Figure 3). It was unclear to us what caused the outcome, especially since anti-LT antibodies were not detected in this mouse. 

 Knowing only STa_A14Q_ toxoid was studied in this study, we realized that this 3xSTa_A14Q_-tmLT may not necessarily be the optimal fusion antigen for ETEC vaccine development. Continuous studies to evaluate STa toxoids and STa-LT toxoid fusions in eliciting neutralizing anti-STa antibodies will help us to identify optimal toxoid fusion antigen(s) for ETEC vaccines development. Nevertheless, results from this study indicated that: 1) LT-STa toxoid fusions are proper antigens to induce protective immunity against ETEC toxins; 2) this STa_A14Q_ toxoid is a better candidate for constructing LT-STa toxoid fusions to elicit neutralizing anti-STa antibodies; and 3) additional copies of STa toxoid enhanced the toxoid fusion for anti-STa immunogenicity. That suggested this 3xSTa_A14Q_-tmLT fusion antigen can be potentially used in vaccine development against ETEC diarrhea. In addition, the approach of fusing multiple copies of a small and poorly immunogenic antigen to further enhance its immunogenicity may be useful in general vaccine development.
